# Embodied Cognition in Performance: The Impact of Michael Chekhov’s Acting Exercises on Affect and Height Perception

**DOI:** 10.3389/fpsyg.2019.02277

**Published:** 2019-10-09

**Authors:** Ana Hedberg Olenina, Eric L. Amazeen, Bonnie Eckard, Jason Papenfuss

**Affiliations:** ^1^School of International Letters and Cultures, Arizona State University, Tempe, AZ, United States; ^2^Department of Psychology, Arizona State University, Tempe, AZ, United States; ^3^Herberger Institute for Design and the Arts, Arizona State University, Tempe, AZ, United States; ^4^School of Sustainability, Arizona State University, Tempe, AZ, United States

**Keywords:** acting, affect, height perception, Michael Chekhov, movement, psychological gesture

## Abstract

Modern embodied approaches to cognitive science overlap with ideas long explored in theater. Performance coaches such as Michael Chekhov have emphasized proprioceptive awareness of movement as a path to attaining psychological states relevant for embodying characters and inhabiting fictional spaces. Yet, the psychology of performance remains scientifically understudied. Experiments, presented in this paper, investigated the effects of three sets of exercises adapted from Chekhov’s influential techniques for actors’ training. Following a continuous physical demonstration and verbal prompts by the actress Bonnie Eckard, 29 participants enacted neutral, expanding, and contracting gestures and attitudes in space. After each set of exercises, the participants’ affect (pleasantness and arousal) and self-perceptions of height were measured. Within the limitations of the study, we measured a significant impact of the exercises on affect: pleasantness increased by 50% after 15 min of expanding exercises and arousal increased by 15% after 15 min of contracting exercises, each relative to the other exercise. Although the exercises produced statistically non-significant changes in the perceived height, there was a significant relation between perceived height and affect, in which perceived height increased with increases in either pleasantness, or arousal. These findings provide a preliminary support for Chekhov’s intuition that expanding and contracting physical actions exert opposite effects on the practitioners’ psychological experience. Further studies are needed to consider a wider range of factors at work in Chekhov’s method and the embodied experience of acting in general.

## Introduction

Much of the work in modern cognitive philosophy could be considered embodied, situated, or enactive (Thompson and Varela, 2001; Chemero, 2009; Clark, 2011; Varela et al., 2017). Embodiment, in particular, is also rapidly becoming a topic of interest in cognitive science (Wilson and Gibbs, 2007; Van Dantzig et al., 2008; Glenberg, 2010; Sulutvedt et al., 2018). The key assumption underlying these approaches is that the mental processes we associate with cognition are fundamentally linked to bodily processes, such as perception and movement (and, by extension, to the environment in which the body is situated). The notion that psychological states are simultaneously bodily (or environmental) states is an idea that has been explored separately in theater. Michael Chekhov (1891–1955), an Oscar-nominated Russian-American actor and theater director, left a legacy of actor training techniques and theoretical reflections emphasizing the importance of proprioceptive sensations of movement to embodying a character and inhabiting a fictional space. The roots of Chekhov’s techniques lie in Konstantin Stanislavsky’s system, which cultivated “a spatial adpositional conceptualization of experience” in the actors, helping them construct “a stable attention field” within which they could interact with each other and the environment during performance (Clare, 2017, p. 43). Initially an actor in Stanislavsky’s theater troupe, Chekhov elaborated his own method based on decades of creative work, self-observation, and pedagogical experience. As his approach became widely accepted in acting schools, performance theorists have explored Chekhov’s acting techniques through the lens of cognitive neuroscience (Blair, 2007; Kemp, 2012; Lutterbie, 2015). Yet, while these scholars have identified intriguing parallels between Chekhov’s insights and current scientific models of the mind, there is not enough empirical data to evaluate the psychological effects of Chekhov’s movement exercises. In the present study, then, we tested the effects of expanding and contracting exercises (adapted from Chekhov, 1953, pp. 63–84) on the psychological experiences of perceived height and affect. As our base condition for comparison, we also measured the participants’ self-perception of height and affect after neutral poses, which preceded the contracting and expanding exercise sets. These neutral poses were intended to focus the participants’ attention on the present moment and signal the beginning of the experimental session.

### Poses, Emotions, and Spatial Self-Awareness

The present study proceeds from the premise that proprioceptive sensations of posture and movement are linked to emotional and cognitive processes (Paillard, 2005, pp. 90, 105). From the neurophysiological standpoint, this connection may be explained by the fact that the limbic system of the brain, involved in the emotional experience and expression, projects diffuse reciprocal pathways into the somatosensory and moto-neurons of the locus coeruleus and the nucleus subcoeruleus in the brainstem, enabling access to the spinal cord (Holstege, 1992, p. 78). According to Holstege(1992, p. 78), the “emotional brain has a great impact on the sensory as well as the motor systems.” For example, in the state of aggression, the limbic system is capable of changing membrane excitability of the neurons, evoking analgesia, while at the same time setting the motor system on high alert (Holstege, 1992, p. 78). The psychological dimension of the interconnected relationship between kinesthetic sensations, emotional states, and cognitive processes has been examined in multiple recent studies. In an experiment by Rahona et al. (2014), clinically depressed patients exhibited a greater persistence of the sad mood when they shook their heads at the sight of pleasant images, as opposed to those who nodded when presented with the same images. Likewise, holding “power poses” – socially recognized as dominant – for as little as 2 min has been shown to increase the subjects’ reported self-confidence and willingness to take risks, in addition to raising the level of adrenaline and lowering the level of the stress hormone cortisol in the saliva (Carney et al., 2010). A more nuanced study, which took into account the participants’ self-evaluation prior to experimental manipulation, found that assuming dominant poses heightened the self-confidence of subjects with preexistent high self-esteem and exacerbated the feeling of low self-worth among those who reported this condition before the experiment (Briñol et al., 2009). While the above studies have focused on the impact of consciously induced gestures and poses on mood, a reverse effect has also been documented: elicited moods influence the character of motor action. In a study by Giraud et al. (2016), involving motion capture and analysis technologies, positive moods were associated with a more impulsive movement signature, and negative moods with tenser, more rigid, and jerkier movement signatures.

Movement has also been shown to influence our perceptions of space. As Paillard (2005, p. 91) explains, motor action frames spatial self-awareness, contributing both to our mental predictions about the outside world and our own, perceived and unconsciously registered, body schema. We process visual stimuli in relation to the concomitant sensations of posture, balance, muscle strain, and fatigue, as well as the experience of our bodily size. For example, the ability to judge distance by sight is influenced by a person’s experience of physical effort. In a series of experiments by Proffitt et al. (2003), individuals wearing a heavy backpack estimated distances as longer compared to subjects without backpacks. The experience of walking on a treadmill while blindfolded produced an aftereffect whereby the subjects not only judged distances to be of greater magnitude, but also walked further away from the initial position when asked to “walk in place,” compared to people who walked on the treadmill with no interruption of their optical flow. In another study, again using backpacks, Bhalla and Proffitt (1999) reported that wearing a heavy backpack (approximately 20% of body weight) impacted the way in which people judged the steepness of a hill in front of them. Participants in the study who wore backpacks reported steeper hill inclinations, both verbally and visually, than participants without. Findings such as these have been interpreted to signify that spatial awareness is influenced by the costs associated with intended and performed actions (Proffitt, 2006, 2008). Several studies have also reported an increase of visual acuity in subjects carrying weights, although gradually making the load heavier does not produce corresponding incremental changes to vision (Gonzalo-Fonrodona and Porras, 2013; Yonemitsu et al., 2017).

Not only has research concluded that proprioceptive sensations of movement influence mood and spatial perception, but these two psychological experiences have been shown to interact, suggesting a complex mediating effect. In a study by Riener et al. (2011), participants’ moods were manipulated before they were asked to estimate geographic slant. The subjects reporting a sad mood were more likely to overestimate the incline of a slope than participants reporting a happy mood. Similarly, a cross-sectional study by Duguid and Goncalo (2012) suggested that individual power was correlated with perceptions of height. During a multi-part experiment, participants’ power was manipulated both experientially and by role-playing. Results suggested that having greater power was associated with smaller estimates of the height of external objects and larger estimates of one’s own height.

### Michael Chekhov’s “Psychological Gesture” Concept

The exercises used in the present experiment were adopted from Michael Chekhov’s description of “psychological gesture” exercises in his book *To the Actor* (1953). Chekhov encouraged his students to approach the role they were working on by assuming a pose that, in their view, expressed the gist of the character’s affects and attitudes. He believed that starting from such a single large gesture or pose, involving the whole body, was an effective way for the actor to coax his or her imagination, discover additional nuances of the character’s mindset, and, in the process, attain an emotional state relevant for the role (Chekhov, 1953, p. 63). This pathway simultaneously allowed for launching a chain of psychophysiological reactions relevant for the role and preventing these processes from spinning out of control (compared, for example, to Konstantin Stanislavsky’s and Lee Strasberg’s method of emotional recall, where the actor had to dwell on personal memories reminiscent of the situation in the dramatic script). Chekhov described his training technique as learning to manipulate the inner “energy” in tandem with the physical expressions of the body, so as to adopt the character’s internal state and render it communicable. What he called “psychological gesture” was a means to “influence, stir, mold and attune your whole inner life to its artistic aims and purposes (Chekhov, 1953, p. 71).”

Like many early 20th-century theorists of performance, Chekhov conceived of human behavior in terms of the dichotomy between willful and subconscious processes, and emphasized movement and posture exercises as a way of initiating autonomous psychophysiological responses that are not subject to the immediate willful command (Sofia, 2014; Lutterbie, 2015). To help actors temporarily merge with the characters they sought to portray, Chekhov laid emphasis on gesture and pose as a foundational stepping stone for developing the contour of the role. The “psychological gestures” used by Chekhov in training actors were symbolic archetypes, or condensed sketches of various characters’ most salient attitudes. Although “psychological gestures” begin as willfully induced, symbolic movements, Chekhov argued that dwelling on the somaesthetic experiences induced by these motor actions leads the actors to embrace the character’s mood and attitudes as their own:

So we may say that the strength of the movement stirs our will power in general; the kind of movement awakens in us a definite corresponding desire, and the quality of the same movement conjures up our feelings (Chekhov, 1953, p. 65).

Chekhov’s technique, then, aims to provide a physical approach to uncovering a character’s emotional state. The text of the play provides the given circumstances, dialogue, relationships, and action of the character that guide the actor to create an appropriate “psychological gesture.” Using the text as a template, Chekhov directed actors to create bold gestures as the first step toward inhabiting the character’s inner life.

In this study, we were not guided by a dramatic text; rather, we appealed to the participants’ imagination to provide motivations for the movement. In this respect, we took inspiration from Chekhov’s stand-alone exercises, not tied to any play (Chekhov, 1953, pp. 63–84), in which various gestures and poses were combined with verbal prompts relevant to the character’s temperament and attitudes. For example, Chekhov associated an energetic thrust of one’s arms upward and a wide stance with a prophet-like figure, characterized by “a fanatical, fiery will,” who is “open to influences from above,” but at the same time “stands firmly on the ground and receives equally strong inspirations from the earthly world” (Chekhov, 1953, p. 68). To create an opposite effect, Chekhov (1953) asked his students to inhabit a character who is “entirely introspective, with no desire to come into contact either with the world above or below, but not necessarily weak” (p. 68). This “brooding” persona prone to self-isolation was portrayed by the bent head, clenched fists, arms pressed close to the body, and unstable, contorted posture suggesting withdrawal and avoidance (Chekhov, 1953, p. 68). It is important to note that Chekhov did not provide precise instructions on the actual movements and poses to be performed: he presented his students only with character traits and some general guidelines on gestures, leaving it up to each person to discover the expression they thought most appropriate. In our experiment, we designed exercises that are closely aligned with Chekhov’s principles, although we modified the wording of the verbal cues as described in the “Materials and Methods” section. Our verbal prompts served to create an interpretative context for the movements and stimulate the participants’ imagination.

Chekhov’s method is routinely used by both professional actors working on new roles, and by novices taking their first steps in acting training. Our study focused on the core principle underlying the creative process of the actor in Chekhov’s system – the possibility of psychological transformation through guided exercise.

### Overview

The present study investigated whether movement exercises, foundational for actors’ training based on Michael Chekhov’s system, impact subjects’ emotional state and perception of their own height. Participants in this experiment performed three sets of exercises based on Chekhov (1953): neutral, expanding, and contracting. A set of neutral exercises, performed at the beginning of the experiment, emphasized natural, relaxed postures and served as a baseline condition. Expanding exercises emphasized outward movement of the limbs and energetic, assertive posture in an imaginary supportive environment while contracting exercises encouraged curling up, withdrawal, and hiding from imagined oppression. Immediately after performing each set of exercises, participants reported their mood and perceived height. Consistent with the idea that psychological states are simultaneously bodily states, we expected to find systematic changes in mood and perceived height following the exercises. Such a demonstration would support the theoretical basis of Chekhov’s method and open the door to further exploration of the actor’s creative process based on modern findings regarding embodied cognition.

## Materials and Methods

### Participants

Twenty-nine undergraduate students (20 female, 9 male) at the Arizona State University participated in this experiment. We have not collected data on their previous experience with acting and various formal techniques of bodily mastery, such as dance, martial arts, or yoga, because Chekhov’s method of actors’ training does not distinguish between professional practitioners and novices (i.e., both beginners and professionals are expected to cultivate proprioceptive awareness and explore new poses that are not tied to codified movement protocols). Each participant was paid $10 upon arrival. Participants ranged in age from 18 to 49 years old (mean = 25.9 years; standard deviation = 7.5 years). The mean height of the participants was 172.8 cm (standard deviation = 10.6 cm; range from 158 to 198 cm).

### Design

**FIGURE 1:**
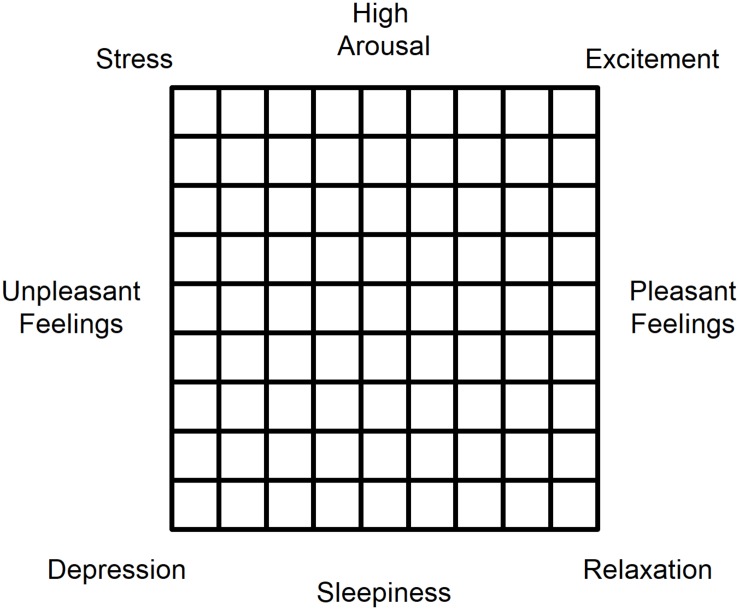
Affect Grid (Russell et al., 1989) used to report Mood. Participants placed an X in the box that corresponded to their current experience of arousal (vertical dimension) and pleasantness (horizontal dimension).

In groups of 5–10 (4 groups total), participants were led through three sets of exercises based on Chekhov (1953): neutral, expanding, and contracting. For two of the groups, the sequence was neutral, expanding, contracting, and for the other two, neutral, contracting, expanding. After each set of exercises, participants completed a survey of affect using an Affect Grid (Russell et al., 1989; see Figure 1) and reported their self-perception of height (Perceived Height) using a line projected on a wall. The Affect Grid provided separate measures of arousal and pleasantness, each on a 9-point scale. Analysis of Variance (ANOVA) was used to evaluate whether mood (arousal and pleasantness) and perceived height changed as a function of exercise. Multiple regression was used to identify whether variations in arousal and pleasantness could predict variations in perceived height.

### Apparatus

The experiment was run in a large room typically used for theater and dance rehearsals. A curtain hung through the middle of the room so that the exercises were done on one side of the curtain and the measurements of mood and perceived height were done on the other. Mood was measured using an Affect Grid (Russell et al., 1989). An Affect Grid (Figure 1) is a 9 × 9 grid of boxes with pleasantness labeled along the horizontal dimension (from unpleasant on the left to pleasant on the right) and arousal labeled on the vertical dimension (from sleepiness on the bottom to aroused on the top). In the four corners of the Grid, the dimensions of pleasantness and arousal intersect, producing four affective states: stress, excitement, depression, and relaxation. The Affect Grid is commonly used to gather reports of mood for self (Cain et al., 2019; Emmerdinger and Kuhbandner, 2019; Finisguerra et al., 2019; Ogawa and Nittono, 2019) and others (Cain et al., 2019; Chen and Whitney, 2019). The advantage for the present experiment is that it allowed for rapid and repeated measures of mood while the participant is engaged in other tasks. Grids were preprinted on individual sheets of paper so that participants would make each report on a clean grid, with no information about their previous responses. Participants were instructed to place a mark in the square that corresponded to their combined experience of arousal and pleasantness at that moment. Perceived height was measured using an electronic version of the methods used by Warren and Whang (1987) and Mark (1987). Participants stood 6.1 m from a wall and matched a line projected onto the wall to their self-perception of height. A projector connected to a computer was used to project a horizontal white line on a black wall. There were no marks on the wall to provide feedback about actual height and all furniture was moved away from the wall. Using a handheld remote, each participant was able to move the line up and down until it matched their self-perception of height. Two measures of height (one with the line starting at the top of the range, 223 cm, and one starting at the bottom, 136 cm) were taken after each set of exercises; the two measures were averaged and used as the perceived height for that condition.

### Procedure

Upon entering the room, the participants’ actual height was measured using a tape measure. For the experiment, participants were led through three sets of exercises, adapted from Chekhov (1953, pp. 62–84). Each set of exercises began by adopting a posture, followed by a set of movements derived from that posture, and finished with movements through the room. The first set, serving as a baseline, was always the Neutral exercises. We started with it in an effort to focus the participants’ attention on the present moment and the inception of the experiment. This set began by standing in a relaxed posture with the feet directly under the hips and arms and hands relaxed by the sides. Then participants were instructed to imagine that the head is a balloon, floating up and lengthening the spine. They then walked around the room in a relaxed and neutral posture. The second and third sets of exercises (expanding and contracting) were performed in a randomized order: in two groups, the sequence was neutral, expanding, contracting, and in the other two, neutral, contracting, expanding. The expanding exercises began by standing in a comfortable posture while imagining a source of energy “radiating” from inside their torso, or an “internal center.” Then participants were instructed to raise their arms, widen their stance, expand their torso, and lengthen their reach and body. They finished this set of exercises by lunging forward in different directions and moving through the room in a way that filled as much space as possible. In the contracting exercise, participants were asked to imagine that the space outside of them was shrinking, and some imaginary outside force was closing in around them. Participants were verbally prompted to push away or retreat from these external energies. The contracting exercises began by adopting a contracted posture in which they made themselves as small as possible while imagining an external force acting upon them. Then participants were instructed to make retreating movements intended to resist or avoid this external force. They finished this set of exercises by moving through the room as if this external force was compressing and slowing them. In each set of exercises, the instructions were general, and participants were encouraged to be creative in how they expressed their movements. Participants were also free to open or close their eyes.

Each set of exercises lasted 15 min. At the end of each 15-min set, an experimenter tapped participants randomly one-by-one to perform the measurements of mood and perceived height. Each of the three times (i.e., after the neutral exercise, after the contracting, and after the expanding exercise), the participants picked up a new, clean Affect Grid to report mood and then moved on to report perceived height. After making these two reports, participants returned to the group exercises and were instructed to continue the exercises until the new set began. The entire procedure lasted approximately 1 h. The Institutional Review Board at Arizona State University approved all elements of the procedure.

## Results

### Actual and Perceived Height

Actual and perceived height data are reported for all participants in Table 1. The mean height of the participants was 172.8 cm (SD = 10.6 cm; range = 159–198 cm). The mean perceived height across all experimental conditions was 178.9 cm (SD = 10.4 cm; range = 161.8–200 cm). Actual and perceived height were significantly correlated, *r*(27) = 0.7, *p* < 0.01.

**TABLE 1:** Actual and perceived height in cm.

**Participant**	**Actual height**	**Neutral**	**Expanding**	**Contracting**
1	172	177.2	180.8	178.1
2	171	173.7	168.8	157.3
3	164	167.1	167.4	168.1
4	168	178.4	168.3	165.2
5	167	181.6	183.5	184.7
6	198	193.3	195.3	198.4
7	164	178.4	172.3	172.8
8	179	179.4	175.0	180.1
9	168	173.7	173.5	175.9
10	182	177.4	185.7	176.4
11	171	182.5	182.5	179.8
12	186	193.1	199.4	187.4
13	185	179.1	183.3	182.3
14	159	175.4	176.9	165.7
15	162	164.4	167.9	161.5
16	171	176.7	174.5	171.8
17	172	174.2	177.6	189.9
18	162	170.5	159.3	162.2
19	171	178.6	178.1	180.1
20	182	194.5	205.0	200.4
21	165	167.4	162.5	155.6
22	162	178.4	165.4	164.4
23	198	192.8	198.7	195.5
24	158	177.4	177.2	180.6
25	169	178.6	181.3	183.0
26	188	179.8	171.8	176.4
27	175	194.8	209.0	196.2
28	176	183.0	190.6	188.2
29	167	179.1	180.3	176.4
Mean	172.8	179.3	179.7	177.7
Standard error	2.0	1.5	2.3	2.3

*Perceived height was measured after each of three sets of exercise. All participants performed the neutral exercises first. Participants 1–15 performed the expanding exercises next while participants 16–29 performed the contracting exercises next.*

The ANOVA of perceived height as a function of exercise is reported in Table 2. The main effect of exercise on perceived height was not significant, *F*(2,56) = 1.53, *p* = 0.23, ${}_{p}^{2}$ = 0.052. Mean perceived height (and standard error) was 179.3 cm (1.4 cm) following the neutral exercise; 177.7 cm (2.3 cm) following the contracting exercise; and 179.7 cm (2.3 cm) following the expanding exercise. Although the effect was not significant, 17 out of the 29 participants reported feeling shorter after the contracting exercises, compared to the expanding exercises.

**TABLE 2:** Analysis of variance results for perceived height.

**Source**	***df***	**Mean square**	***F***	***p***	**${}_{p}^{2}$**
Exercise	2	31.96	1.53	0.225	0.05
	56	20.84			

### Affect

**FIGURE 2:**
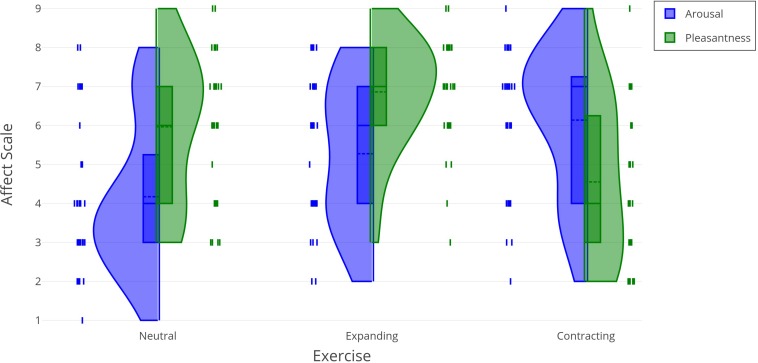
Arousal and pleasantness as a function of exercise.

**FIGURE 3:**
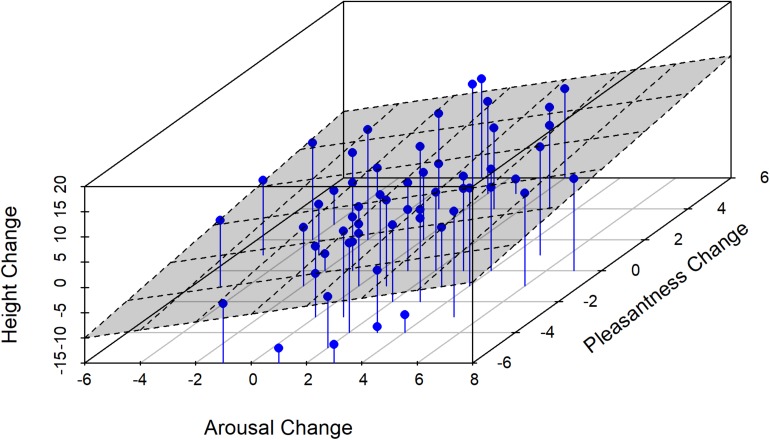
A 3D linear regression plot, showing correlations between self-perceived height, pleasantness, and arousal.

Reported arousal and pleasantness as a function of exercise for all participants are reported in Table 3. Violin plots in Figure 2 show the probability density correlating changes in the reported arousal and pleasantness with each type of exercise. The ANOVA results are presented in Table 4. Overall, participants felt more pleasant than aroused, *F*(1, 28) = 4.83, *p* = 0.036, ${}_{p}^{2}$ = 0.15. There was also a main effect of exercise, in which the combined arousal and pleasantness were greater in the expanding condition, *F*(2, 56) = 5.61, *p* = 0.006, ${}_{p}^{2}$ = 0.17. However, these main effects were superseded by the significant interaction between mood and exercise, *F*(2, 56) = 13.17, *p* < 0.001, ${}_{p}^{2}$ = 0.32. The data in Figure 2 suggests that this interaction resulted from the expanding and contracting exercises having different effects on each measure of affect. Pairwise *t*-tests comparing arousal and pleasantness across the expanding and contracting exercise conditions revealed significant effects for each. Arousal was greater after performing the contracting exercises, compared to the expanding exercises, *t*(28) = 2.60, *p* = 0.015; pleasantness was greater after performing the expanding exercises, compared to the contracting exercises, *t*(28) = 5.08, *p* < 0.001. Figure 3 visualizes the correlations between the self-perceived height, arousal, and pleasantness as a 3D linear regression plot.

**TABLE 3:** Reports of arousal and pleasantness following each set of exercises.

	**Neutral**		**Expanding**		**Contracting**	
**Participant**	**Arousal**	**Pleasantness**	**Arousal**	**Pleasantness**	**Arousal**	**Pleasantness**
1	5	8	8	8	8	7
2	7	6	4	7	8	3
3	4	5	6	3	6	2
4	7	3	3	8	2	2
5	3	8	4	7	7	7
6	3	9	4	6	7	4
7	5	3	4	7	6	7
8	3	6	2	6	6	6
9	8	4	3	5	4	5
10	6	4	5	4	4	4
11	4	6	4	6	4	5
12	2	7	7	8	8	2
13	7	6	3	8	3	5
14	3	4	6	8	9	2
15	2	8	8	7	8	4
16	2	7	6	8	4	2
17	3	4	7	7	6	2
18	7	3	4	6	3	6
19	3	7	2	6	7	7
20	3	6	7	9	8	3
21	8	4	4	6	6	4
22	4	7	8	7	7	3
23	2	7	4	8	4	3
24	1	9	6	8	8	9
25	3	8	7	9	7	7
26	4	7	7	5	7	6
27	4	7	8	8	7	5
28	4	3	6	7	7	7
29	4	7	6	7	7	3
Mean	4.2	6.0	5.3	6.9	6.1	4.6
Standard error	0.4	0.4	0.4	0.3	0.3	0.4

*All participants performed the neutral exercises first. Participants 1–15 performed the expanding exercises next while participants 16–29 performed the contracting exercises next.*

**TABLE 4:** Analysis of variance results for affect.

**Source**	***df***	**Mean square**	***F***	***p***	**$\eta_{p}^{2}$**
Exercise	2	15.47	5.61	0.006	0.17
	56	2.76			
Mood	1	15.54	4.83	0.036	0.15
	28	3.22			
Exercise × mood	2	52.02	13.17	0.000	0.32
	56	3.95			

### Perceived Height as a Function of Affect

Because there was variability in each measure not attributable to conditions, we sought a direct effect of mood on perceived height. To normalize the data to reflect changes within a participant and not across participants, the value of each measure (perceived height, arousal, and pleasantness) after the neutral exercises was subtracted from the values after the expanding and contracting exercises. These data (ΔPerceived Height, ΔArousal, and ΔPleasantness), then, indicate how much each measure changed relative to neutral. A multiple regression analysis (see Tables 5, 6) revealed a significant effect of ΔArousal and ΔPleasantness on ΔPerceived Height, *R* = 0.403, *F*(2, 57) = 5.321, *p* = 0.008. The coefficients on both predictors were significant: ΔArousal, *b* = 0.79, *t* = 2.65, *p* = 0.011; ΔPleasantness, *b* = 0.68, *t* = 2.58, *p* = 0.013. The resulting regression equation is,

**TABLE 5:** Analysis of variance results for the multiple regression of ΔPerceived Heaviness on ΔArousal and ΔPleasantness.

**Model**	***df***	**Mean square**	***R***	***F***	***p***
Regression	2	176.65	0.403	5.32	0.008
Residual	55	33.20			
Total	57				

**TABLE 6:** Coefficients for the multiple regression of ΔPerceived Heaviness on ΔArousal and ΔPleasantness.

**Model**	***b***	**Standard error**	**β**	***t***	***p***
Constant	−1.22	0.80		−1.53	0.132
ΔArousal	0.79	0.30	0.34	2.65	0.011
ΔPleasantness	0.68	0.26	0.33	2.58	0.013

Δ⁢Perceived Height=-1.22+0.79×Δ⁢Arousal+0.68 (1)

×Δ⁢Pleasantness

Equation 1 reveals that increases in both arousal and pleasantness were associated with increases in perceived height. As participants felt more aroused and more pleasant, they felt taller. The reason that that there was no significant effect of exercise on perceived height, therefore, may have been that each exercise condition produced an increase in one dimension of mood and a decrease in the other, possibly canceling out the mean changes in perceived height.

## Discussion

The present study investigated whether the movement exercises used in Michael Chekhov’s method produce noticeable effects on the participants’ psychological experience, specifically, on their affective state and their perception of their own height. Chekhov’s method for training actors was predicated on the assumption that movement influenced psychological experience, attuning the subject to the role. The present study was motivated to test for the specific psychological effects based on recent research supporting an embodied, situated, and enactive perspective on psychology (Thompson and Varela, 2001; Chemero, 2009; Clark, 2011; Varela et al., 2017). Participants in this experiment performed three sets of exercises adapted from Chekhov (1953): neutral, expanding, and contracting. Following each set of exercises, participants reported their perceived height and affect. Consistent with expectations, these exercises induced changes in affective states. In comparison to their self-evaluations after the neutral exercises, the participants reported feeling more positive and aroused after the expanding exercises and more negative and aroused after the contracting exercises. It was possible to note that overall, the expanding and contracting exercises we observed tended to shift the participants’ affect up and down along the diagonal running from “relaxation” (low arousal, high pleasure) to “stress” (high arousal, low pleasure) on the Affect Grid. On the other hand, the results for the self-perception of height did not reveal a statistically significant change after either of the three interventions. However, we have observed a strong correlation between a taller perception of height and increased arousal and pleasantness. The latter finding opens up a pathway for future research by suggesting that a more likely potential for shifting the subjects’ self-perception of height could be found in experiments, capable of manipulating the participants’ mood along the diagonal running from “depression” (low arousal and low pleasantness) to “excitement” (high arousal and high pleasantness) on the Affect Grid.

Very few previous studies have examined the effect of embodied or situated cognition on the perception of one’s own height. Duguid and Goncalo (2012) established a positive correlation between the individuals’ experience of power and self-perceived height, suggesting a general connection between perceived height and mood. In the present experiment, despite the lack of significant differences in perceived height across conditions, we found a significant relation between perceived height and the two dimensions of affect (arousal and pleasantness). Increases in both arousal and pleasantness were associated with increases in perceived height.

Taken together, our results suggest that the contracting and expanding acting exercises developed by Chekhov (1953) produce systematic changes in the psychological experience, specifically insofar as the practitioners’ self-perceived arousal vs. sluggishness, and pleasant vs. unpleasant feelings are concerned. These results provide preliminary support to Chekhov’s intuition that physically performing specific “psychological gestures” triggers specific inner responses from the practitioners.

In reporting these preliminary results, this study’s limitations must be acknowledged, so as to mark the specificity of our findings and point to future studies that may help further elucidate the factors shaping the subjects’ experience during Chekhov’s “psychological gesture” exercises. We have used the neutral exercises as our base condition for our comparisons, with no measurements of affect taken prior to the beginning of the experiment. Our study also did not involve a control group. Objections may be raised as to whether any form of physio-mental exercise could produce the same effects as the contracting and expanding exercises we have adapted from Chekhov. In anticipating this objection, we point to the opposite trends in self-reported mood that we found after the expanding and contracting exercises, which suggests that specific types of movement routines, coupled with appropriate verbal instructions, tend to evoke specific inner experiences. Future studies may compare the subjects’ self-perceptions of mood and height after Chekhov’s acting exercises and after purely physical exercises, not accompanied by verbal prompts that endow actions with semantic connotations.

### Movement and Affect in Context

An important feature of artistic performance is that it typically occurs within the context of a character, a narrative, or some situational circumstances. Michael Chekhov designed his exercises to help actors discover embodied nuances of their roles within a specific dramatic text. In fact, the purpose of Chekhov’s “psychological gesture” was to help actors capture complex context with simple archetypal gestures. Although we stripped the exercises of a specific narrative or character, verbal instructions allowed participants to generate their own imagined context. Thus, our verbal prompts, such as a request to imagine interacting with an “external force” while doing the exercises, could be considered situational circumstances. It is essential, therefore, to consider the role of context when evaluating the individuals’ psychological experiences occurring in tandem with their physical performances.

A seminal study on the “power poses” by Carney et al. (2010) has linked open, expansive postures (expressions of power) and closed, contractive postures (expressions of powerlessness) to specific neuroendocrine changes, further implying that a given set of poses will consistently produce the same psychological effect. The exercises used in the present study bear resemblance to dominant and submissive poses in Carney et al. (2010). In a critical review, Bavel (2013) pointed out that the linking of “power poses” to the increased self-confidence may not be as context-free as Carney et al. (2010) suggest. Other studies, according to Bavel (2013), demonstrate the importance of social context in which the experimental manipulations are taking place. Thus, Cesario and McDonald (2013) showed that the experience of dominance and submissiveness induced by expansive and constrictive poses occurred only within an interpersonal context, which endowed gestures with specific meanings. For example, holding an expansive pose while imagining being frisked by police resulted in less powerful behavior (Cesario and McDonald, 2013). As Laird and Bresler (1992) explain, the individual’s self-perception is influenced both by the proprioceptive signals of posture and situational awareness.

Although the present study assessed the participants’ self-perception of mood and height in subjective, ego-centric terms, we do not want to conclude that the psychological effects we observed are context-free. While the verbal instructions accompanying the exercises did not cue the participants to pay attention to their standing vis-à-vis others, they involved abstract metaphors of “supportive energy” in case of expansive gestures and “oppressing energy” in case of contracting ones. We found that in these circumstances, the expanding exercises produced a positive and relaxed mood, as they were coupled with verbal prompts to imagine “supportive energy” spreading out from the torso and filling up the body. However, it is possible to imagine a different scenario, in which the actor were performing expanding exercised to inhabit a character who is asserting his victory over an opponent. In this case, it is likely that the actor would follow up with a primal scream, suggesting an aroused rather than relaxed mood. This counterexample, however, does not diminish the value of research on the role of proprioceptive sensations of posture and gestures in mood regulation. Rather, it prompts us to acknowledge the role of situational circumstances, which impose an interpretative framework on the person’s perceptions of their own bodily state.

### Conclusion: Acting as Embodiment. Chekhov’s Theory in Light of the Present Study

Chekhov’s exploration of the “psychological gesture” anticipated modern embodied, situated, and enactive approaches to psychology. Whether he worked with beginner actors or seasoned professionals, Chekhov encouraged his students to enact their character’s state of mind and attitudes in a specific scene through large gestures involving the entire body. By concentrating on the proprioceptive sensations of this pose, Chekhov’s actor achieved the psychological state useful for approximating the character’s internal emotional landscape.

Our study confirms Chekhov’s predictions about the interplay between the sensations of bodily pose and affective state. We demonstrated that expanding and contracting gestures and postures, coupled with verbal metaphors describing the situation, produce systematic effects on the subjects’ mood: expansive movements result in higher pleasure, while closing in on oneself brings about higher arousal. Neither type of exercise appeared to significantly impact the subjects’ self-perception of height. However, a correlation between the increased sense of tallness and rises in the self-estimates of arousal and pleasantness that we have observed opens up a potentially productive new research pathway, by suggesting a hypothesis that exercises inducing low arousal and low pleasantness (i.e., the state of depression as defined by the Affect Grid) would more likely bring about lowered estimates of one’s own height.

Key aspects of Chekhov’s training method were thus transformed in our study into testable hypotheses with the aim of deriving conclusions about general psychological mechanisms, relevant both inside and outside the actors’ studio. We believe that a quantificational inquiry of this kind does not undermine the artistic dimension of Chekhov’s legacy; nor does our study exhaust the full implications of Chekhov’s exploration of embodiment. We share the performance theorist John Lutterbie’s sense that cognitive psychology helps reveal the significance of Chekhov’s “psychological gesture” in the creative process and provide a stronger foundation for this method of actor training without reducing the full spectrum of the artist’s insights and observations to one rigid formula (Lutterbie, 2015, p. 96).

Acting in general remains an underexplored area for psychology, compared to music and visual arts (Goldstein, 2009). This relative scarcity of scientific studies is surprising, given the fact that many prominent 20th-century directors, whose work stands at the foundation of contemporary theater, have explicitly drawn on various trends of psychological and neurophysiological research (Roach, 1985; Pitches, 2005; Sofia, 2014; Sirotkina and Smith, 2017). Recent scientific studies have considered professional performance longitudinally, from the point of view of personal psychology in its developmental and social dimensions. Thus Goldstein (2009) has pointed out that empathy and emotional adaptability are childhood precursors of acting talent, which get mobilized in theater work in adulthood, while Thomson and Jaque (2011, 2012) have revealed potentially pathological tendencies of fantasy proneness, dissociation, and emotional vulnerability in professional actors. The immediate experience of acting – the process of imaginary transformation, which simultaneously involves the actor’s body and mind – has been studied much less, but this is precisely the area where the cross-disciplinary dialogue between performers and cognitive psychologists has the greatest promise of illuminating the general human mechanisms of perception and mood regulation.

## Data Availability Statement

All datasets generated for this study are included in the manuscript/supplementary files.

## Ethics Statement

The studies involving human participants were reviewed and approved by the IRB, Arizona State University (exemption granted; case no. STUDY00005403). The patients/participants provided their written informed consent to participate in this study.

## Author Contributions

AO conceptualized the overall approach of the study, did bibliographic research, and wrote the Introduction, Discussion, and Conclusion sections of the manuscript. EA carried out the data analysis and wrote the Materials and Methods and Results sections. BE created the exercise script on the basis of Michael Chekhov’s book, led the exercise sessions for the subjects, and provided consultations on the ways in which Chekhov’s teachings are employed in acting schools. JP contributed to the design of experiment materials, did bibliographic research, and created data graphs for the manuscript.

## Conflict of Interest

The authors declare that the research was conducted in the absence of any commercial or financial relationships that could be construed as a potential conflict of interest.
